# Effectiveness of Behaviorally Informed Letters on Health Insurance Marketplace Enrollment: A Randomized Clinical Trial

**DOI:** 10.1001/jamahealthforum.2022.0034

**Published:** 2022-03-04

**Authors:** David Yokum, Daniel J. Hopkins, Andrew Feher, Elana Safran, Joshua Peck

**Affiliations:** 1 Brown University, Providence, Rhode Island; 2 University of Pennsylvania, Philadelphia; 3 Covered California, Sacramento; 4 Office of Evaluation Sciences, US General Services Administration, Washington, DC; 5 Indpendent researcher, Washington, DC

## Abstract

**Importance:**

Every year during the open enrollment period, hundreds of thousands of individuals across the Affordable Care Act marketplaces begin the enrollment process but fail to complete it, thereby resulting in coverage gaps or going uninsured.

**Objective:**

To investigate if low-cost ($0.55 per person) letters can increase health insurance enrollment.

**Design, Setting, and Participants:**

This intent-to-treat randomized clinical trial was conducted during the final 2 weeks of the 2015 open enrollment period among the 37 states on the HealthCare.gov platform. The trial targeted 744 510 individuals who started the enrollment process but had yet to complete it. Data were analyzed from January through August 2021.

**Interventions:**

Study participants were randomized to either a no-letter control group or to 1 of 8 letter variants that drew on evidence from the behavioral sciences about what motivates individuals to take action.

**Main Outcomes and Measures:**

The primary outcome was the health insurance enrollment rate at the end of the open enrollment period.

**Results:**

Of the 744 510 individuals (mean [SD] age, 41.9 [19.6] years; 53.9% women), 136 122 (18.3%) were in the control group and 608 388 (81.7%) were in the treatment group. Most lived in Medicaid nonexpansion states (72.7%), and a plurality were between 30 and 50 years old (41.0%). For race and ethnicity, 3.0% self-identified as Asian, 14.0% as Black, 5.1% as Hispanic, 39.8% as non-Hispanic White, and 38.2% as other or unknown. By the end of the open enrollment period, 4.0% of the control group enrolled in health insurance coverage. Comparatively, the enrollment rate in the pooled treatment group was 4.3%, which demonstrated an increase of 0.3 percentage points (95% CI, 0.2-0.4 percentage points; *P*<.001), yielding 1753 marginal enrollments. Letters that used action language caused larger enrollment effects, particularly among Black individuals (increase of 1.6 percentage points; 95% CI, 0.6-2.7 percentage points; *P* = .003) and Hispanic individuals (increase of 1.5 percentage points; 95% CI, 0.0-3.0 percentage points; *P* = .046) in Medicaid expansion states.

**Conclusions and Relevance:**

This randomized clinical trial shows that letters designed with best practices from the behavioral sciences literature were a low-cost way to increase health insurance enrollment in the Affordable Care Act marketplaces. More research is needed to understand what messages are most effective amid the recently passed American Rescue Plan.

**Trial Registration:**

ClinicalTrials.gov Identifier: NCT05010395

## Key Points

**Question:**

How much do behaviorally informed letters increase health insurance enrollment?

**Findings:**

In this randomized clinical trial that included 744 510 individuals on the HealthCare.gov platform during the final 2 weeks of the 2015 open enrollment period, use of a single behaviorally informed letter caused a statistically significant increase in health insurance enrollment. Letters that used action language caused larger effects, particularly among Black and Hispanic individuals in Medicaid expansion states.

**Meaning:**

Policy makers can use low-cost letter nudges to increase enrollment across Affordable Care Act marketplaces.

## Teaser

This randomized clinical trial investigates if low-cost behaviorally informed letters can increase health insurance enrollment across Affordable Care Act marketplaces.

## Introduction

Through the expansion of Medicaid eligibility and the creation of health insurance marketplaces, the Affordable Care Act (ACA) has helped reduce the uninsured rate to record lows.^1^ But every year during the open enrollment period, hundreds of thousands of individuals who initiate the enrollment process fail to complete it. Gaps in coverage or prolonged bouts of being uninsured cause disruptions in access to care and medication, increased financial strain, higher rates of medical debt, and lower levels of self-reported health.^2,3,4^ Thus, identifying effective strategies to help individuals who have started the enrollment process obtain health insurance remains a priority for policy makers.^5^

Barriers to health insurance take-up are well documented and include cost, application complexity, procrastination, a lack of awareness about available options, choice overload, and inertia.^6,7,8,9^ A growing body of research seeks to understand how different forms of outreach can overcome these barriers to increase enrollment. A recent set of nonexperimental studies, for example, found an association between the volume of health insurance TV advertisements and reductions in the uninsured rate, as well as in ACA marketplace enrollment.^10,11^ And randomized clinical trials (RCTs) have found that nudges using emails, letters, and telephone outreach increased health insurance take-up.^12,13,14^

We build on this empirical evidence in 2 principal ways. First, in contrast with single-state RCTs, the present randomized intervention includes all 37 states that used the HealthCare.gov platform in 2015. The inclusion of multiple states is important within the context of the ACA, where states’ policy decisions, such as Medicaid expansion, affect the cost of coverage and, in turn, whether individuals with low incomes can afford health insurance. Second, in lieu of nonexperimental studies that draw on self-reported survey data, we use administrative data paired with an RCT.

In the final weeks of the 2015 open enrollment period, we conducted an intent-to-treat RCT using behaviorally informed letters to increase health insurance enrollment among individuals who started the enrollment process but had yet to finish it. With 37 states and more than 744 500 individuals, this is, to our knowledge, one of the largest RCTs conducted on the ACA marketplaces to date, though there has been a larger RCT targeting tax filers who owed a positive penalty amount owing to the individual mandate.^14^ Because letters are a low-cost option to reach a large number of uninsured individuals, they could represent a valuable tool for ACA marketplace administrators seeking to increase enrollment.

## Methods

### Study Design and Participants

This study used a parallel 9-arm design with 8 letter variants, each designed based on different insights from the behavioral science literature. The ninth arm was a hold-out control group that did not receive any letter, enabling us to measure the effect of receiving any letter as well as to tease apart the relative effect of the different behavioral features. The study followed the Consolidated Standards of Reporting Trials (CONSORT) reporting guidelines, its protocol was approved by the California Health and Human Services Agency’s institutional review board (Supplement 1), and it was overseen by an interdisciplinary team at the Office of Evaluations Sciences in the US General Services Administration and the Centers for Medicare & Medicaid Services Office of Communications in the US Department of Health and Human Services (HHS).

Study participants were English-speaking individuals who, as of mid-January 2015, had visited HealthCare.gov and registered for a user account but not yet enrolled in an insurance plan. We chose mid-January as the cutoff to maximize the number of individuals eligible for the intervention while also leaving enough time to complete the requisite implementation steps so letters would arrive during the final 2 weeks of the open enrollment period.

Of the 811 795 individuals initially included, 18% were assigned to the no-letter control group, while the remaining 82% were assigned to 1 of 8 letter treatments (Table 1; see eAppendix in Supplement 2 for copies of each of the letters used). The sample size and randomization scheme were chosen because HHS wanted to treat as many consumers as possible before the open enrollment period ended, while also learning about the effects of letter outreach.

**Table 1.** Characteristics of Study Participants at Baseline

Covariate	Treatment arm, %								
	Control (no letter)	Basic letter	Action letter	Action, implementation letter	Action, implementation, picture letter	Norm letter	Norm, pledge letter	Loss aversion letter	Kitchen sink (all features) letter
No.	136 122	75 828	75 993	75 990	76 039	76 164	76 125	76 086	76 163
Race and ethnicity									
Asian	3.0	3.0	2.9	2.9	2.9	2.9	3.0	3.1	2.9
Black	14.1	14.0	13.9	13.9	14.0	13.9	14.1	13.8	14.0
Hispanic	5.1	5.0	5.3	5.0	5.2	4.9	5.1	5.1	5.1
Non-Hispanic White	39.7	39.6	39.9	39.9	39.3	40.0	39.9	39.9	40.0
Other/unknown^a^	38.0	38.4	38.0	38.4	38.6	37.9	37.9	38.1	38.1
Medicaid status									
Expansion state	27.3	27.2	27.3	27.2	27.3	27.3	27.3	27.5	27.4
Nonexpansion state	72.7	72.8	72.7	72.8	72.7	72.7	72.7	72.5	72.6
Age, y									
<30	23.5	23.1	23.6	23.2	23.1	23.5	23.2	23.3	23.5
30-50	41.3	41.1	40.8	41.4	40.8	41.1	40.9	40.8	40.8
>50	35.3	35.8	35.6	35.4	36.1	35.4	35.9	36.0	35.7

^a^ Other/unknown corresponds to individuals who opted not to provide a specific race or ethnicity when applying for health insurance.

### Intervention

Individuals in the treatment arms were assigned to receive letters at the beginning of February 2015, giving them approximately 2 weeks to complete their enrollment. The 8 letters varied behavioral dynamics, including action language, an implementation intention prompt, a picture of then–chief executive officer of the marketplace Kevin Counihan, social norm messaging, a pledge, and loss aversion. These messages drew on evidence from prior randomized interventions that suggested these appeals would induce individuals to take action.^15,16,17^ All letters included the same core information about the benefits of enrolling, the February 15 sign-up deadline, the HealthCare.gov website, and the call center telephone number. The trial ended on February 15, 2015, because that marked the end of the open enrollment period, as well as the call-to-action date in the letters. Data were analyzed from January through August 2021.

### Randomization

Randomization was conducted by the first study author (D.Y.) based on user identification numbers using the sample function and a fixed seed in R, version 3.0.2 (R Foundation). The list with assignments was given to a contractor who mailed the letters.

### Data Sources and Primary Outcome

**Figure 1.**
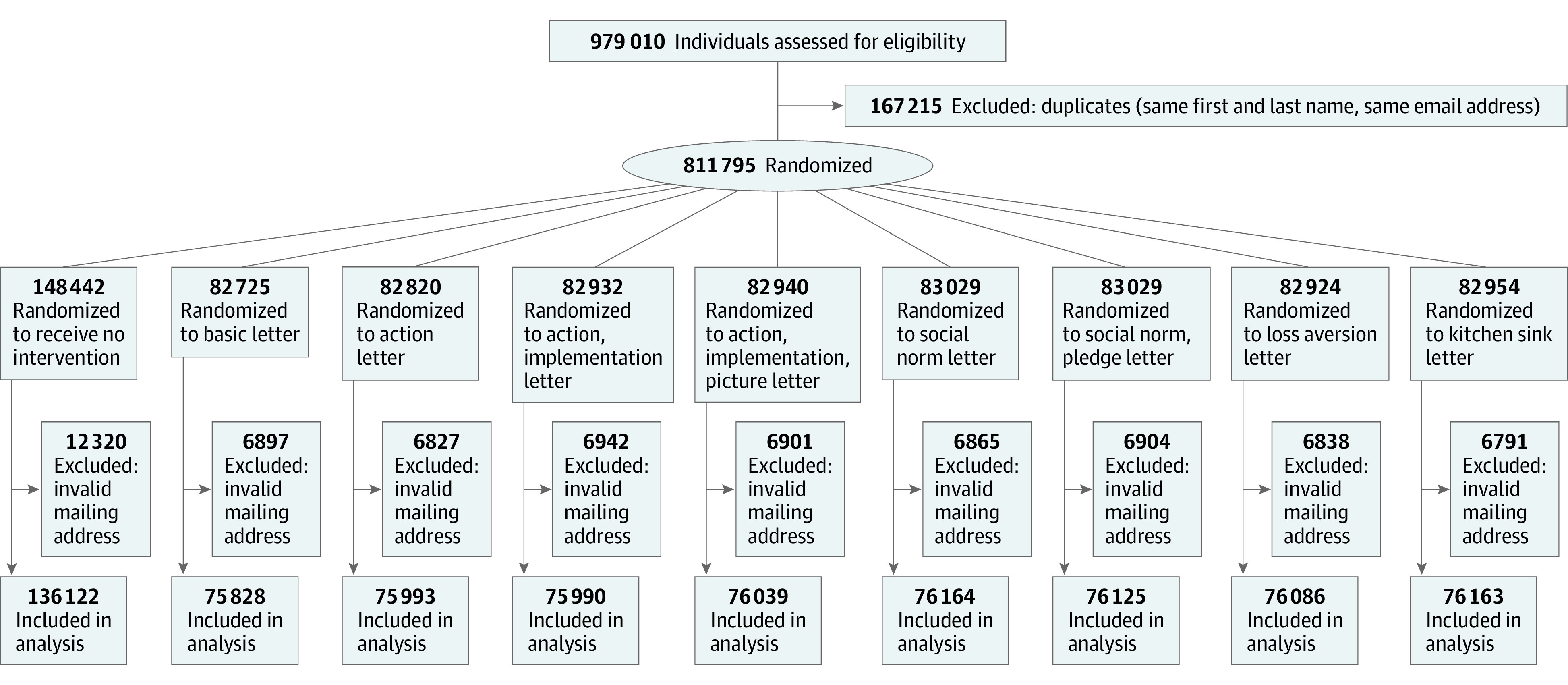
CONSORT Flow Diagram of Included Individuals

At the end of the open enrollment period, we obtained administrative enrollment data from HHS that identified the primary outcome: whether an individual enrolled in an ACA plan on or before the February 15 open enrollment deadline. The sample size for analysis included 744 510 individuals because 67 285 individuals provided invalid mailing addresses, leaving them unable to receive letters or unable to enroll through the HealthCare.gov platform (Figure 1). In eTables 1 and 2 in Supplement 2, we show that the rate of invalid mailing addresses was approximately 8% across arms and was not correlated with treatment assignment. The administrative data also included pretreatment characteristics that we used to assess the validity of the random assignment and for stratification analyses, including self-reported race and ethnicity, state of residence, and age bracket.

### Statistical Analysis

To estimate the effect of the letters overall and by subgroup, we used linear regression models with robust standard errors to account for heteroscedasticity. Data were analyzed using Stata, version 15 (StataCorp), and statistical significance was defined as a 2-sided *P* < .05.

## Results

**Figure 2.**
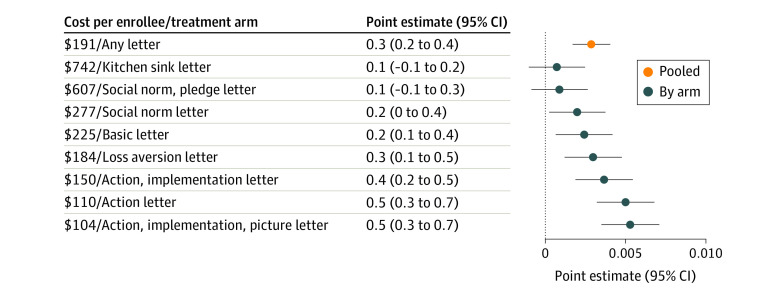
Effect of Letter on Affordable Care Act Enrollment Rate Pooled, by Arm, and Cost per Enrollee Each point represents the average effect in percentage points. Error bars denote 95% CIs.

Of the 744 510 individuals included in the analysis, the mean (SD) age was 41.9 (19.6) years, and 53.9% were women. By the end of the open enrollment period, 4.0% of the control group had enrolled in ACA health insurance. Relative to the control group, assignment to a letter increased enrollment by a statistically significant 0.3 percentage points (95% CI, 0.2-0.4 percentage points; *P*<.001), which represents a 7% increase above the control group mean and amounts to 1753 marginal enrollments (Figure 2). Each letter cost $0.55 per individual, yielding an overall cost per new enrollee of $191.

However, not all letters were equally effective; of the 8 letter variants, 2—the social norm with a pledge and the “kitchen sink” with all features—did not increase enrollment relative to the control group by a statistically significant amount. Letters that used action language (ie, treatment arms 2, 3, and 4) yielded the largest effects, increasing enrollment by 0.5 percentage points (95% CI, 0.3-0.6 percentage points; *P*<.001). If the best-performing letter—the variant that used action language, an implementation prompt, and a picture—was implemented at scale, this would have translated to 3228 marginal enrollees and a cost per new enrollee of $104.

In exploratory analyses, we detected statistically significant differences across most subgroups, except for individuals younger than 30 years and those who did not provide a race and/or ethnicity when applying. The point estimate for Asian adults is substantively large (0.6 percentage points) but imprecisely estimated owing to a relatively small sample size (Table 2). The largest enrollment increase was among Hispanic adults, which was an increase of 0.7 percentage points (95% CI, 0.1-1.3 percentage points; *P* = .02), or 14%.

**Table 2.** Absolute and Relative Changes in Health Insurance Enrollment by Consumer Characteristics

Subgroup	No.	Control group mean	Effect of any letter (SE)	Change, %
Total	744 510	0.04	0.003 (0.001)^a^	7.5
Medicaid expansion				
Yes	203 290	0.046	0.003 (0.001)^b^	6.7
No	541 220	0.038	0.003 (0.001)^a^	7.4
Characteristics of the head of household				
Race and ethnicity				
Asian	22 103	0.061	0.006 (0.004)	10.6
Black	104 047	0.044	0.004 (0.002)^a^	10.2
Hispanic	37 708	0.050	0.007 (0.003)^b^	14.2
Non-Hispanic White	296 418	0.051	0.003 (0.001)^a^	6.1
Other/unknown^c^	284 234	0.025	0.001 (0.001)	4.8
Age, y				
<30	173 729	0.047	0.002 (0.001)	3.6
30-50	305 401	0.045	0.003 (0.001)^a^	7.0
>50	265 380	0.030	0.004 (0.001)^a^	11.6

^a^ *P* < .01.

^b^ *P* < .05.

^c^ Other/unknown corresponds to individuals who opted not to provide a specific race or ethnicity when applying for health insurance.

**Figure 3.**
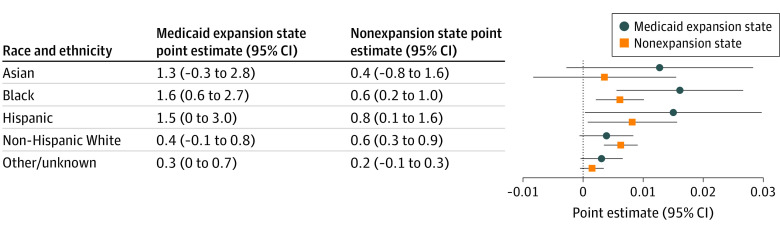
Effect of Action Letters on Affordable Care Act Enrollment Rate by Race and Ethnicity and States’ Medicaid Expansion Status Each row represents the average effect of a letter in percentage points. Error bars denote 95% CIs.

We additionally examined the effect of action letters by race and ethnicity and states’ Medicaid expansion status (Figure 3). In expansion states, the effect of action letters was especially pronounced among racial and ethnic minorities, causing enrollment increases of 1.6 percentage points (95% CI, 0.6-2.7 percentage points; *P* = .003) among Black adults, 1.3 percentage points (95% CI, −0.3 to 2.8 percentage points; *P* = .11) among Asian adults, and 1.5 percentage points (95% CI, 0.0-3.0 percentage points; *P* = .046) among Hispanic adults, a pattern consistent with cost as an enrollment barrier for those living in nonexpansion states where premiums tend to be higher and subsidies are inaccessible to residents with the lowest income.^18^

## Discussion

In this RCT, during the final weeks of the 2015 open enrollment period, we found that low-cost ($0.55 per person) behaviorally informed letters targeting individuals on the HealthCare.gov platform led to statistically significant increases in health insurance enrollment, yielding 1753 marginal enrollments. Letters that used action language, emphasizing that only minimal, marginal effort was required (ie, included the phrase “You’re almost done”), were most effective. Subgroup analyses demonstrated that the largest enrollment increases occurred among Black and Hispanic adults in Medicaid expansion states. These results suggest that low-cost reminders could be a useful tool for ACA marketplace administrators seeking to help individuals obtain coverage prior to sign-up deadlines.

In terms of cost-effectiveness, this study’s $191 cost per marginal enrollment compares favorably with other reported estimates, which range from less than $100 to as high as $1000.^19^ The observed effect sizes are similar in relative terms to those found in the 2 prior studies^12,13^ evaluating mailers to increase health insurance enrollment in single-state contexts. In California, letters sent to applicants of Covered California (that state’s marketplace), who were determined eligible but had not yet selected a plan, led to a 1.3 percentage point (16%) increase over a base enrollment rate of 8.1%. In Oregon, a suite of outreach activities (mail, email, and telephone reminders) led to a 3.5 percentage point (10%) increase in Medicaid enrollment over a base rate of 38%. The absolute percentage point differences across these interventions could be owing to premium costs (or the absence thereof in the case of Medicaid enrollment), the amount of time individuals had to complete the call to action, differences in the duration over which outcomes are measured, or numerous other factors.

While the present randomized intervention was conducted in 2015, recent surveys of uninsured adults indicate that more than 50% still lack awareness of marketplaces and subsidies to make health insurance more affordable, pointing to the need for continued outreach.^20^ With the March 2021 passage of the American Rescue Plan, which expands subsidies for people at every income level through 2022, it will be important for marketplaces to test a variety of different messages to identify what resonates with prospective enrollees and to avoid deploying ineffective outreach strategies.

### Limitations

This research design is based on random assignment, which provides a strong basis for causal inference, but the study is not without limitations. First, owing to operational timelines, letters were only printed in English and sent to households with a written language preference of English; thus, we do not measure effects among harder-to-reach non-English–speaking households.^21^ But because the intervention sought to address commonly cited barriers to enrollment, including procrastination and lack of awareness about the deadline or how to get help, we would expect the reminder letters to have comparable effects among Spanish-speaking individuals. Ultimately, though, this is an empirical question, and we encourage marketplace administrators to draw on promising experimental evidence—including in-language personalized telephone assistance, which has been found to considerably increase marketplace enrollment—during future open enrollment cycles.^22^ Second, because race and ethnicity are optional questions on the ACA application, they are subject to missingness. In the present study, 62% of individuals answered these application questions. Third, letters were sent to individuals who took the initial steps of beginning the enrollment process. Outreach efforts to the uninsured who have not interacted with marketplaces could have different effects. Finally, the letters arrived during the last 2 weeks of the open enrollment period, and we received anecdotal reports of letters arriving in mailboxes after the February 15 deadline. Thus, the estimated treatment effect potentially represents an underestimate because late letters could not have affected enrollment prior to the deadline, and it is possible that letters sent near the start of the open enrollment period could have had different effects.

## Conclusions

In this RCT, we found that a low-cost letter, targeting individuals who took the first steps toward enrolling in ACA marketplace coverage but stopped short of selecting a plan, caused statistically significant and meaningful increases in ACA health insurance enrollment. From 2017 to 2020, enrollment in the ACA marketplaces declined from 12.2 million to 11.4 million in part because of a reduction in marketing and advertising. As the Biden administration seeks to expand coverage, particularly among racial and ethnic minorities hard hit by the COVID-19 pandemic, this study provides evidence that low-cost outreach—especially messages informed by the behavioral sciences—could help increase ACA marketplace enrollment.

## Notes

Supplement 1. Trial Protocol

Supplement 2.
eTable 1.

eTable 2.
**eAppendix.** Intervention Materials

Supplement 3. Data Sharing Statement
