# Supplementary material for: Effectiveness of Behaviorally Informed Letters on Health Insurance Marketplace Enrollment: A Randomized Clinical Trial
Source: JAMA Health Forum. 2022 Mar 4;3(3):e220034. doi: 10.1001/jamahealthforum.2022.0034 (PMC8903125; doi:10.1001/jamahealthforum.2022.0034)
Supplement: Supplement 1. — Trial Protocol [file jamahealthforum-e220034-s001.pdf]

ClinicalTrials.gov Protocol Registration and Results System (PRS) Receipt
Release Date: August 10, 2021

ClinicalTrials.gov ID: NCT05010395

Study Identification

Unique Protocol ID: 02152015

Brief Title: Increasing Health Insurance Enrollment Among Uninsured Americans

Official Title: Increasing Health Insurance Enrollment Among Uninsured Americans

Secondary IDs:

Study Status

Record Verification: August 2021

Overall Status: Completed

Study Start: January 15, 2015 [Actual]

Primary Completion: February 15, 2015 [Actual]

Study Completion: June 1, 2015 [Actual]

Sponsor/Collaborators

Sponsor: Office of Evaluation Sciences

Responsible Party: Principal Investigator
Investigator: David Yokum [dyokum]
Official Title: Director of The Policy Lab
Affiliation: Brown University

Collaborators:

Oversight

U.S. FDA-regulated Drug: No

U.S. FDA-regulated Device: No

U.S. FDA IND/IDE: No

Human Subjects Review: Board Status: Approved
Approval Number: 2021-149
Board Name: California Health and Human Services Institutional Review Board
Board Affiliation:
Phone: 916-326-3661
Email: lucila.martinez@oshpd.ca.gov
Address:

Data Monitoring: No

FDA Regulated Intervention: No

Study Description

Brief Summary: This randomized evaluation used behaviorally-informed letters to increase health insurance take-up on the Affordable Care Act (ACA) Marketplaces.

Detailed Description: During Open Enrollment Periods for the ACA Marketplaces, qualifying individuals can purchase health insurance plans through the Federal Health Insurance Marketplace (FHIM). As of early February 2015, many people had visited HealthCare.gov and started an online account, but not yet selected a plan. The Department of Health and Human Services (HHS), in collaboration with the Office of Evaluation Sciences (OES), developed and sent letters to assist these individuals with completing their enrollment before the deadline.

To evaluate the effect of the letters, the team used a randomized controlled trial.

Conditions

Conditions: Health Behavior

Keywords: health insurance enrollment

Study Design

Study Type: Interventional

Primary Purpose: Other

Study Phase: N/A

Interventional Study Model: Parallel Assignment

Number of Arms: 9

Masking: None (Open Label)

Allocation: Randomized

Enrollment: 811795 [Actual]

Arms and Interventions

Arms	Assigned Interventions
No Intervention: Control<br>Individuals in this arm did not receive a letter.	
Experimental: Arm 1: Basic Letter<br>Individuals received a letter with information about the benefits of enrolling, the February 15th sign-up deadline, the HealthCare.gov website, and the call center phone number.	Behavioral: Letter intervention<br>Letter intervention
Experimental: Arm 2: Action<br>Individuals in this arm received a letter that emphasized only minimal marginal effort is required; and used adjectives and verbs connoting action (e.g., “almost done,” “quick,” “act now,” and “fast”).	Behavioral: Letter intervention<br>Letter intervention
Experimental: Arm 3: Action, Implementation<br>Individuals in this arm received a letter similar to Arm 2, but with the addition of a calendar that draws attention to the February 15th deadline; and provided	Behavioral: Letter intervention<br>Letter intervention

Arms	Assigned Interventions
fill-in blanks in which the recipient can write the planned month, day, and time when they intend to enroll.	
Experimental: Arm 4: Action, Implementation, Picture<br>Individuals in this arm received a letter similar to Arms 2 and 3, but with the addition of an image of then HealthCare.gov Chief Executive Officer Kevin Counihan	Behavioral: Letter intervention<br>Letter intervention
Experimental: Arm 5: Norm<br>Individuals in this arm received a letter that included the following social norm sentence: “Americans are enrolling but you haven’t joined them.”	Behavioral: Letter intervention<br>Letter intervention
Experimental: Arm 6: Norm, Pledge<br>Individuals in this arm received a letter that included a statement, to be checked in agreement, that “I pledge to Get Covered at Healthcare.gov.”	Behavioral: Letter intervention<br>Letter intervention
Experimental: Arm 7: Loss Aversion<br>Individuals in this arm received a letter that warned “You risk paying a fee of $325 or 2% of your income—whichever is higher.”	Behavioral: Letter intervention<br>Letter intervention
Experimental: Arm 8: Kitchen Sink<br>Individuals in this arm received a letter that includes all behavioral dynamics except for the pledge (due to space limitations).	Behavioral: Letter intervention<br>Letter intervention

Outcome Measures

Primary Outcome Measure:

1. Percent of individuals enrolling in an ACA plan by the end of the Open Enrollment period.
   Percent of individuals enrolling in an ACA plan during the observation period.

   [Time Frame: Two weeks]

Eligibility

Minimum Age:

Maximum Age:

Sex: All

Gender Based: No

Accepts Healthy Volunteers: No

Criteria: Inclusion Criteria:

- Those with an English language preference; those who had not enrolled in an ACA plan as of early February 2015

Exclusion Criteria:

- Those with a Spanish language preference

This intervention was a program evaluation and did not go through Human Subjects Protection Review.

Contacts/Locations

Central Contact Person: Andrew Feher, PhD
Email: andrew.feher@covered.ca.gov

Central Contact Backup: Elana Safran, MPP
Email: elana.safran@gsa.gov

Study Officials: David Yokum, PhD
Study Principal Investigator
Brown University

Locations:

IPDSharing

Plan to Share IPD: Yes
Deidentified replication data will be posted to OSF upon manuscript acceptance.

Supporting Information:
Study Protocol
Analytic Code

Time Frame:
Access Criteria:
URL: https://osf.io/pb256/

References

Citations:

Links:

Available IPD/Information:
