# Supplementary material for: Effectiveness of Behaviorally Informed Letters on Health Insurance Marketplace Enrollment: A Randomized Clinical Trial
Source: JAMA Health Forum. 2022 Mar 4;3(3):e220034. doi: 10.1001/jamahealthforum.2022.0034 (PMC8903125; doi:10.1001/jamahealthforum.2022.0034)
Supplement: Supplement 2. — eTable 1. eTable 2. eAppendix. Intervention Materials [file jamahealthforum-e220034-s002.pdf]

Supplemental Online Content

Yokum D, Hopkins DJ, Feher A, Safran E, Peck J. Effectiveness of behaviorally informed letters on health insurance marketplace enrollment: a randomized clinical trial. *JAMA Health Forum*. 2022;3(3):e220034. doi:10.1001/jamahealthforum.2022.0034

eTable 1.

eTable 2.

eAppendix. Intervention Materials

This supplemental material has been provided by the authors to give readers additional information about their work.

© 2022 Yokum D et al. *JAMA Health Forum.*

Attrition Analysis

As noted in the main text, 67,285 individuals provided invalid mailing addresses. We exclude these individuals from our intent-to-treat analysis because they were either unable to receive letters or unable to enroll through the Healthcare.gov platform. Below we present evidence that the rate of invalid mailing addresses was uncorrelated with treatment assignment.

eTable 1.

	(1)
DV: Invalid Mailing Address	Pooled
Treatment	-0.000
	(0.001)
Constant	0.083***
	(0.001)
Observations	811,795

Robust standard errors in parentheses
*** p<0.01, ** p<0.05, * p<0.1

eTable 2.

	(1)
DV: Invalid Mailing Address	Arm
Basic	-0.001
	(0.001)
Action	0.000
	(0.001)
Action/Implement	-0.000
	(0.001)
Action/Implement/Pic	0.000
	(0.001)
Social Norm	-0.001
	(0.001)
Social Norm/Pledge	0.001
	(0.001)
Loss Aversion	-0.001
	(0.001)
Kitchen Sink	0.000
	(0.001)
Constant	0.083***
	(0.001)
Observations	811,795

Robust standard errors in parentheses
*** p<0.01, ** p<0.05, * p<0.1

© 2022 Yokum D et al. *JAMA Health Forum.*

Intervention Materials

Basic Letter

Health Insurance Marketplace

DEPARTMENT OF HEALTH AND HUMAN SERVICES
465 INDUSTRIAL BOULEVARD
LONDON, KENTUCKY 40750-0001

[Date]
[Address of Primary Contact]

Dear [First name last name]:

It's time to come back to HealthCare.gov and complete your enrollment for 2015 health coverage. If you've completed your enrollment already, that's great – you can skip this letter and refer to the information your health plan sends you.

If you haven't finished enrolling, it's not too late to get high-quality health coverage that meets your needs. Plans available in the Health Insurance Marketplace provide essential health benefits like **free preventive care** and most people qualify for **financial help**. Marketplace coverage can also protect you from unexpected medical costs in the future.

Millions of Americans are already benefiting from the **quality, affordable health coverage** available to them through the Health Insurance Marketplace. We want you to join them!

Open Enrollment for 2015 ends on February 15, 2015, for Marketplace coverage starting March 1. We don't want you to miss this opportunity for health coverage. Return to HealthCare.gov today!

For more information, visit HealthCare.gov or call the Marketplace Call Center toll-free at 1-800-318-2596. TTY users should call 1-855-889-4325.

© 2022 Yokum D et al. *JAMA Health Forum.*

Action Letter

Health Insurance Marketplace

Dear [First name]:

You're *almost* done at HealthCare.gov — only an extra step or two needed.

You've started your online enrollment for 2015 health insurance coverage, so you already know the benefits: affordable premiums, guaranteed coverage, free preventive services, and most people also qualify for financial help. And if you have any questions, quick help is available on the website or at **1-800-318-2596**. (TTY users should call 1-855-889-4325.)

But you need to act now to finish. The final deadline, February 15th, is approaching fast. **Visit HealthCare.gov** right away and complete your enrollment.

(If you've completed your enrollment already, that's great — you beat us to the punch!)

Sincerely,

Health Insurance Marketplace
Department of Health and Human Services

© 2022 Yokum D et al. *JAMA Health Forum.*

Action Implementation Letter

Health Insurance Marketplace

Dear [First name]:

You're *almost* done at HealthCare.gov — only an extra step or two needed.

You've started your online enrollment for 2015 health insurance coverage, so you already know the benefits: affordable premiums, guaranteed coverage, free preventive services, and most people qualify for financial help. And if you have any questions, quick help is available on the website or at **1-800-318-2596**. (TTY users should call 1-855-889-4325.)

But you need to act now to finish. The final deadline, February 15th, is approaching fast. **Visit HealthCare.gov** right away and complete your enrollment.

(If you've completed your enrollment already, that's great — you beat us to the punch!)

Sincerely,

Health Insurance Marketplace
Department of Health and Human Services

P.S. Don't have time this second to finish? Make a plan for when you'll act, and go stick it on your refrigerator so you don't forget!

- -

January 2015

S	M	T	W	T	F	S
				1	2	3
4	5	6	7	8	9	10
11	12	13	14	15	16	17
18	19	20	21	22	23	24
25	26	27	28	29	30	31

February 2015

S	M	T	W	T	F	S
1	2	3	4	5	6	7
8	9	10	11	12	13	14
15						

I will complete my enrollment at HealthCare.gov on:

[ ] , [ ] [ ] at [ ]

(name of day) (month) (day #) (time)

© 2022 Yokum D et al. *JAMA Health Forum.*

Action, Implementation, Picture Letter

Health Insurance Marketplace

Dear [First name]:

You're *almost* done at HealthCare.gov — only an extra step or two needed.

You've started your online enrollment for 2015 health insurance coverage, so you already know the benefits: affordable premiums, guaranteed coverage, free preventive services, and most people qualify for financial help. And if you have any questions, quick help is available on the website or at **1-800-318-2596**. (TTY users should call 1-855-889-4325.)

But you need to act now to finish. The final deadline, February 15th, is approaching fast. **Visit HealthCare.gov** right away and complete your enrollment.

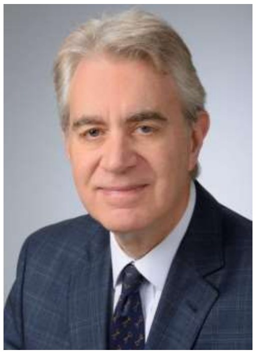

(If you've completed your enrollment already, that's great — you beat us to the punch!)

Sincerely,

Kevin Counihan
Chief Executive Officer
Health Insurance Marketplace

P.S. Don't have time this second to finish? Make a plan for when you'll act, and go stick it on your refrigerator so you don't forget!

January 2015

S	M	T	W	T	F	S
				1	2	3
4	5	6	7	8	9	10
11	12	13	14	15	16	17
18	19	20	21	22	23	24
25	26	27	28	29	30	31

February 2015

S	M	T	W	T	F	S
1	2	3	4	5	6	7
8	9	10	11	12	13	14
15						

I will complete my enrollment at HealthCare.gov on:

________, ________ ____ **at** ______

(name of day) (month) (day #) (time)

© 2022 Yokum D et al. *JAMA Health Forum.*

Social Norm Letter

Health Insurance Marketplace

Dear [First name]:

Americans are enrolling at HealthCare.gov — but you haven't joined them.

Millions of Americans have already found health coverage at HealthCare.gov. They know the benefits: affordable premiums, guaranteed coverage, free preventive services, and most people qualify for financial help. And for people with questions, they took advantage of the help available on the website and at **1-800-318-2596**. (TTY users called 1-855-889-4325.)

But you haven't joined them yet. The final deadline, February 15th, is approaching, and many people have already enrolled. Join your fellow Americans on HealthCare.gov to complete your enrollment.

(If you've completed your enrollment since we mailed this letter, that's great – you're already part of the HealthCare.gov family!)

Sincerely,

Health Insurance Marketplace
Department of Health and Human Services

© 2022 Yokum D et al. *JAMA Health Forum.*

Social Norm and Pledge Letter

Health Insurance Marketplace

Dear [First name]:

Americans are enrolling at HealthCare.gov — but you haven't joined them.

Millions of Americans have already found coverage at HealthCare.gov. They know the benefits: affordable premiums, guaranteed coverage, free preventive services, and most people qualify for financial help. And for people with questions, they took advantage of the help available on the website and at **1-800-318-2596**. (TTY users called 1-855-889-4325.)

But you haven't joined them yet. The final deadline, February 15th, is approaching, and many people have already enrolled. Join your fellow Americans on HealthCare.gov to complete your enrollment.

(If you've completed your enrollment since we mailed this letter, that's great – you're already part of the HealthCare.gov family!)

Sincerely,

Health Insurance Marketplace
Department of Health and Human Services

P.S. Join us! Check the pledge below, and stick it on your refrigerator so you don't forget. Show others that you value your health.

- -

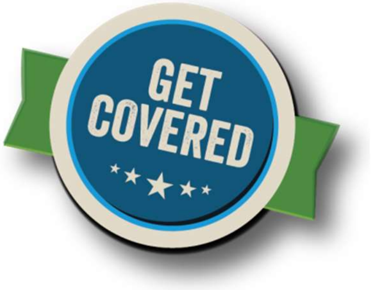

☐ I pledge to **Get Covered** at HealthCare.gov and join the millions already doing their part to keep America healthy.

© 2022 Yokum D et al. *JAMA Health Forum.*

Loss Aversion Letter

Health Insurance Marketplace

Dear [First name]:

You risk paying a fee of $325 or 2% of your income — whichever is higher.

If you don't get health coverage for 2015, you risk paying a fee with your taxes — either $325 per person or 2% of your income (whichever is higher). To avoid this fee, complete your enrollment at HealthCare.gov, where you can also take advantage of affordable premiums, guaranteed coverage, and free preventive services; most people also qualify for financial help. If you have questions, help is available on the website and at **1-800-318-2596**. (TTY users should call 1-855-889-4325.)

Avoid the fee. The final deadline, February 15th, is approaching. Visit HealthCare.gov to complete your enrollment.

(If you've completed your enrollment since we mailed this letter, that's great – you've already avoided the potential fee for going without coverage!)

Sincerely,

Health Insurance Marketplace
Department of Health and Human Services

© 2022 Yokum D et al. *JAMA Health Forum.*

Kitchen Sink Letter

Health Insurance Marketplace

Dear [First name]:

You're *almost* done at HealthCare.gov — but a little more is needed, or else risk a $325 fee.

You've started your online application for 2015 health insurance coverage, so you already know the benefits: affordable premiums, guaranteed coverage, free preventive services, and most people qualify for financial help. If you have any questions, quick help is available on the website or at **1-800-318-2596**. (TTY users should call 1-855-889-4325.)

But you need to act now. The final deadline, February 15th, is approaching fast. If you don't get health coverage for 2015, you **risk paying a fee** with your taxes — either $325 or 2% of your income (whichever is higher).

(If you've completed your enrollment already, that's great — you beat us to the punch!)

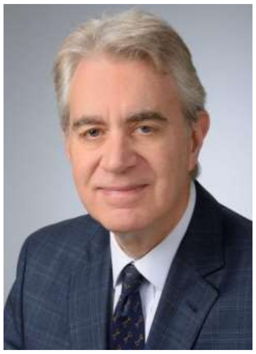

Sincerely,

Kevin Counihan
Chief Executive Officer
Health Insurance Marketplace

P.S. Don't have time this second to finish? Make a plan for when you'll act, and go stick it on your refrigerator so you don't forget!

January 2015

S	M	T	W	T	F	S
				1	2	3
4	5	6	7	8	9	10
11	12	13	14	15	16	17
18	19	20	21	22	23	24
25	26	27	28	29	30	31

February 2015

S	M	T	W	T	F	S
1	2	3	4	5	6	7
8	9	10	11	12	13	14
15						

I will complete my enrollment at HealthCare.gov on:

[ ] , [ ] [ ] at [ ]

(name of day) (month) (day #) (time)

© 2022 Yokum D et al. *JAMA Health Forum.*
