# Supplementary material for: Effectiveness of Behaviorally Informed Letters on Health Insurance Marketplace Enrollment: A Randomized Clinical Trial
Source: JAMA Health Forum. 2022 Mar 4;3(3):e220034. doi: 10.1001/jamahealthforum.2022.0034 (PMC8903125; doi:10.1001/jamahealthforum.2022.0034)
Supplement: Supplement 3. — Data Sharing Statement [file jamahealthforum-e220034-s003.pdf]

Data Sharing Statement

Yokum. Effectiveness of Behaviorally Informed Letters on Health Insurance Marketplace Enrollment. *JAMA Health Forum*. Published March 04, 2022.
doi:10.1001/jamahealthforum.2022.0034

Data
Data available: Yes
Data types: Deidentified participant data
How to access data: https://osf.io/pb256/
When available: With publication

Supporting Documents
Document types: None

Additional Information
Who can access the data: Anyone requesting the data.
Types of analyses: For any purpose.
Mechanisms of data availability: Without investigator support.
